# Selections

**Published:** 1892-09

**Authors:** 


*Secretary.*

### Selections.

### DENTISTS IN THE ARMY AND NAVY.

[We are indebted to the *Southern Dental Journal* for republishing a paper on this subject by F. Y. Clark, M.D., D.D.S., of Savannah, Georgia, and read at the Southern Dental Association, April, 1870. As this matter has recently been under discussion, the abstracts we make will be read, we think, with interest, especially the historical portion. The position of a community without a dentist is vividly portrayed.—Ed.]

Jefferson Davis, when Secretary of War to the United States made an effort to employ dentists in the army and navy, and no doubt would have succeeded had he remained in office. From this time, until after the commencement of the war, we know of no further effort being made. Shut in as we were from the North and the rest of the world, we knew little of what was going on without. When the steamer "Water Witch" was captured and the prisoners brought to Savannah, we learned there was a dentist among the number, and that he had quite a supply of material. From this we were led to believe that our profession was recognized by the government, and that dentists were appointed in the army and navy. Shortly after, however, when General Sherman and his hundred thousand men entered Savannah, we were by practical demonstration better informed. From daylight until dark our dental offices were besieged. The cry was relief from present suffering, "Do something for my teeth that will keep them from aching." We remember one fellow coming to us a few days after having one or two nerves destroyed in his teeth, who wished, as he said, "to buy a good smart quantity of that white, creamy stuff;" for, said he, "when the boys get on the march, and have the toothache, I can get their last dollar with it."

But this was not the only applicant for dental material. Dental instruments were in great demand, and a dental depot would have done a smashing business in Savannah for a few days at that time. Now, would there have been such a demand had there not been a necessity? The innumerable quantity of broken teeth and fractured jaws, produced by bungling instruments and unfamiliar hands, along with stories of rheumatic and neuralgic suffering, caused by exposed pulps and diseased teeth, which we were obliged to listen to during the sojourn of the army in Savannah, were disheartening; and we determined then, should an opportunity ever occur, to contribute our mite towards a remedy.

We cannot but think our general medical director was remiss in his duty in this respect, but not as much so as the members of our own profession, whose duty it was to properly bring the matter before him. At the North almost every State had its society. The American Association and American Convention were in full operation, besides three or four dental colleges, and as many dental journals; yet, with all this array of strength and talent, we hear of little or no effort being made for the end in view. We believe, had the subject been properly brought to the notice of the general medical director, and, with his aid before Congress, by some one of our societies, that dental appointments would have been made in the army and navy, and that the good developed by such appointments would, before now, have produced full recognition on the part of our government as a necessity, and that to-day American dentistry would be occupying a much more elevated position in the eyes of the world than it does.

With us in the South it was very different. We had no dental society or journal, and, owing to the irregularity of the mails and stringency of conscription, we could not act collectively, but had to do what we could individually. As the war went on, and the call for men was renewed and the conscription increased to fifty, we were thinned to almost a corporal's guard. Many towns of several thousand inhabitants had no dentist. This state of affairs wrought a change little thought of. Officers and government employees, including their families, found the inconvenience and suffering too great to be endured, and their appeals became so numerous and urgent that the surgeon-general was obliged to recommend the detailing of dentists for many towns and hospitals. The hospital practice was productive of so much good that the appointment of a dentist to each regiment was strongly urged as a means of increasing the active duty-list of the army. The following is an extract from a memorial which was presented about that time:

"Our own experience with soldiers, in and around Richmond during the last year, in connection with the statements of some of the most intelligent physicians and officers in the service, fully convinces us that out of every one hundred men sent to the hospital, or those on the sick-list,—exclusive of those wounded in battle,—five, at least, can be traced, directly or indirectly, to some derangement of the teeth that might have been remedied in a few minutes or hours. Thus we have, of one hundred thousand men, five thousand unnecessarily off duty. Now, this five thousand in every hundred thousand might be returned to active duty, or prevented from leaving it, by the appointment of a few dentists, say one to every army division."

This, no doubt, would have had its effect, and appointments would have been made, but the end came, and the project was lost with "the lost cause."

Now, is it not humiliating to our nation and profession to be obliged to acknowledge that the English, with their better teeth and worse dentistry, have exercised more humanity in this matter than we have? Just think of one hundred thousand men, as in the case of the army of General Sherman between Atlanta and Savannah, exposed for months to all kinds of weather, and imagine the amount of suffering they must have endured owing to the want of a few dentists! The army of Washington cannot be argued against this, for then our teeth were much better, and no more what they are now than was the practice of dentistry. Again, it cannot be argued that as the war is over the necessity is past, for every now and then we hear of war-vessels leaving for some distant port, with from three to five hundred men, who are away for months and years without ever seeing a dentist.

We all know how and wherein the army and navy could be benefited by the appointment of dentists, but it is no easy matter to convince the appointing power. No doubt, could we make them look on the broken teeth and fractured jaws, and force them to listen to the stories of suffering above mentioned, and, better still, experience a little of the pain there alluded to, away from the means of relief, it would not be long before this vacuum in our army and navy would be filled. But as we cannot do this, we must do what is next best,—we must keep on telling them until they will believe us. No military school, no army post or station, no naval ship, or foreign port where ships are stationed, should be without a dentist. Our sailors and soldiers are no more able to pay for dental operations than they are for medical attention; therefore, if we value their services, we should, as a humane and enlightened nation, value their lives. In many distant and foreign ports our sailors and representatives are no better off than at sea. Take the ports of Japan, for instance, where the native surgeons extract teeth by giving them a thorough malleting on both sides until they are loose enough to be removed. The field for good to our nation and profession in such ports is extensive. The advanced state of dentistry would necessarily (as has been the case in Paris with Evans and others) bring us in contact with the officials and higher classes of the country; and we would thus reach influential ears that are now closed to us. But the most direct and greatest good would be to our sailors and representatives stationed there, for they would thus be within reach of innumerable advantages which the advanced state of dentistry offers. To oblige the army and navy surgeons to fill this vacuum is wrong, it is inhuman; for with their medical education only they are little better prepared to perform any dental operation than the patients they attempt to relieve. No medical college has made any attempt to educate their students in this specialty for the last twenty years; they have given it over to the dental schools, where it properly belongs.

